# Association between Perioperative Blood Transfusions and Clinical Outcomes in Patients Undergoing Bladder Cancer Surgery: A Systematic Review and Meta-Analysis Study

**DOI:** 10.1155/2016/9876394

**Published:** 2016-01-31

**Authors:** Juan P. Cata, Javier Lasala, Greg Pratt, Lei Feng, Jay B. Shah

**Affiliations:** ^1^Department of Anesthesiology and Perioperative Medicine, The University of Texas MD Anderson Cancer Center, Houston, TX 77030, USA; ^2^Anesthesiology and Surgical Oncology Research Group, Houston, TX 77030, USA; ^3^Biostatistics, The University of Texas MD Anderson Cancer Center, Houston, TX 77030, USA; ^4^Medical Library, The University of Texas MD Anderson Cancer Center, Houston, TX 77030, USA; ^5^Urology, The University of Texas MD Anderson Cancer Center, Houston, TX 77030, USA

## Abstract

*Background*. Perioperative blood transfusions are associated with poor survival in patients with solid tumors including bladder cancer.* Objective*. To investigate the impact of perioperative blood transfusions on oncological outcomes after radical cystectomy.* Design*. Systematic review and meta-analysis.* Setting and Participants*. Adult patients who underwent radical cystectomy for bladder cancer.* Intervention*. Packed red blood cells transfusion during or after radical cystectomy for bladder cancer.* Outcome Measurements and Statistical Analysis*. Recurrence-free survival (RFS), cancer-specific survival (CSS), and overall survival (OS). We calculated the pooled hazard ratio (HR) estimates and 95% confidence intervals by random and fixed effects models.* Results and Limitation*. Eight, seven, and five studies were included in the OS, CSS, and RFS analysis, respectively. Blood transfusions were associated with 27%, 29%, and 12% reduction in OS, CSS, and RFS, respectively. A sensitivity analysis supported the association. This study has several limitations; however the main problem is that it included only retrospective studies.* Conclusions*. Perioperative BT may be associated with reduced RFS, CSS, and OS in patients undergoing RC for BC. A randomized controlled study is needed to determine the causality between the administration of blood transfusions and bladder cancer recurrence.

## 1. Introduction

Bladder cancer (BC) is the ninth most common malignancy in the United States with 74,000 new cases and 16,000 deaths estimated for 2015 [1]. Since radical cystectomy (RC) is the standard treatment for clinically localized muscle-invasive bladder cancer many patients undergo surgery with curative intention [2]. Unfortunately, though the majority of patients are rendered disease-free after surgery, a significant proportion go on to develop BC recurrence and to ultimately succumb to the disease. For patients undergoing cancer surgery, there has been recent interest in identifying perioperative factors that may modulate recurrence and cancer-specific survival after surgery. It has been suggested that perioperative blood transfusion (BT) may be one such factor [3–5].

Blood transfusions represent the top five most frequently overused therapeutic procedures in the United States [6, 7]. Unfortunately, a clinically significant number of patients (30–75%) with BC receive blood products during and after RC [8–10]. Although BTs can be life-saving in some clinical perioperative circumstances, there are adverse events associated with their administration including transfusion-related immune suppression (TRIM) [11]. TRIM is one proposed mechanism by which BTs may be linked to poor oncologic outcomes [11]. Several retrospective studies have demonstrated that perioperative BTs are independently predictive of poor survival in patients with bladder cancer [12–14]. A meta-analysis by Wang and colleagues demonstrated the association between BTs and decreased recurrence-free survival (RFS) and overall survival (OS) [15]. However, three recent studies that included more than 6,500 patients in aggregate were recently published and are not part of that meta-analysis [16–18].

We sought to assess the impact of BT on cancer-related outcomes and mortality in patients who had RC for muscle-invasive bladder cancer. We conducted a systematic review of the literature and meta-analysis to test for an association between perioperative BTs and recurrence-free, cancer-specific, and overall survival in patients undergoing RC.

## 2. Material and Methods

### 2.1. Search Methods for Identification of Studies

We searched Ovid MEDLINE and EMBASE, PubMed, Cochrane Library, and the ClinicalTrials.gov databases from inception to June 2015, with no limits of language or publication type. To identify additional studies, we also searched the 2010–2015 meeting abstracts of the American Society of Clinical Oncology, the American Urological Association, and the European Association of Urology. Database search strategies included controlled vocabulary (e.g., Medical Subject Headings) and keyword terms to find studies addressing perioperative transfusions or related procedures (such as blood salvage or hemodilution) of whole blood or blood components in bladder cancer patients. Outcomes sought by the search strategies included blood loss (intraoperatively or postoperatively), cancer-specific outcomes (e.g., recurrence, metastasis, and disease progression), and survival. All searches were performed by a medical librarian (Greg Pratt) who has contributed to more than 50 systematic reviews and meta-analyses.

### 2.2. Data Collection and Analysis

The primary outcomes of interest were recurrence-free survival, cancer-specific survival, and overall survival. We defined a perioperative BT as any amount of pRBC within one month before and one month after RC. We included randomized controlled trials (RCTs), prospective cohorts, and retrospective studies that evaluated the impact on any (allogeneic versus autologous versus intraoperative recovered “cell saved”) packed red blood cells (pRBCs) in patients with BC who underwent RC. We excluded studies considering patients with distant metastases at surgery; those in which recurrence-free survival, cancer-specific survival, or overall survival were not indicated; and abstracts or poster presentations. Quality assessment of the included studies was performed using the Ottawa-Newcastle scale [19]. Studies with a score of 6 or lower in the Ottawa-Newcastle scale were also excluded from any statistical analysis [19]. For studies with overlapping patient populations, only the most recent publication was used. For multicenter studies, data were analyzed separately by center.

We calculated the pooled hazard ratio (HR) estimates and 95% confidence intervals by random effects model using the method of DerSimonian and Laird (D + L). To derive pooled estimates, the D + L method calculates weights by taking the inverse of a combination of within-study and between-study variability, which provides a larger variance compared with the variance produced from fixed effects analyses and thus wider confidence intervals.

Cochran's *Q*-test was used to test the null hypothesis of no significant heterogeneity across studies. Cochran's *Q*-statistic follows *χ* ^2^ distribution with (*k* − 1) degrees of freedom, where *k* is the number of studies. *I* ^2^ or the percentage of variation in the measures of association across studies due to heterogeneity was also calculated. *I* ^2^ is the equivalent to the quantity of Cochran's *Q* minus its degrees of freedom divided by Cochran's *Q*, or *I* ^2^ = (*Q* − df)/*Q*. The value of *I* ^2^ ranges between 0% and 100%, where 0% indicates no observed heterogeneity and larger values indicate increasing heterogeneity. The summary effect measure on hazard ratio for intraoperative transfusion on the time-to-event endpoints (overall survival, cancer-specific survival, and recurrence-free survival) was obtained. Lastly, a sensitivity analysis was conducted to test whether the results of the meta-analysis were sensitive to restrictions on any of the included studies.

A *p* value < 0.05 was considered statistically significant. All statistical analyses were performed using R software (Version 3.0.2, the R Foundation for Statistical Computing).

## 3. Results

### 3.1. Description of Studies

**Figure 1:**
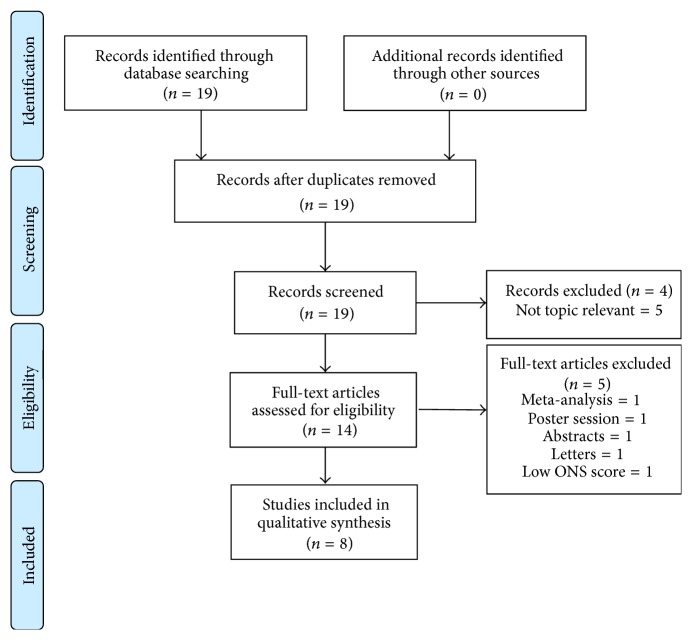
Flow diagram.

The initial search identified 14 potential studies that underwent full review (Figure 1). Of these, 6 studies were excluded and 8 studies were included in the analysis. All of them were retrospective and published between 2012 and 2013. Abel's study included data from 2 different institutions; thus the 2 substudies were considered separately for statistical analysis. The mean quality score of the included studies was 7.44 ± 0.72. Only 5 studies clearly stated that patients were transfused with allogeneic blood; the remaining studies did not specify the type of blood. The leukoreduced status of the blood units was not clarified in any of the included studies. Two studies differentiated between intra- and postoperative blood transfusion and found that patients transfused intraoperatively but not postoperatively had worse survival [10, 17].

### 3.2. Overall Mortality

**Figure 2:**
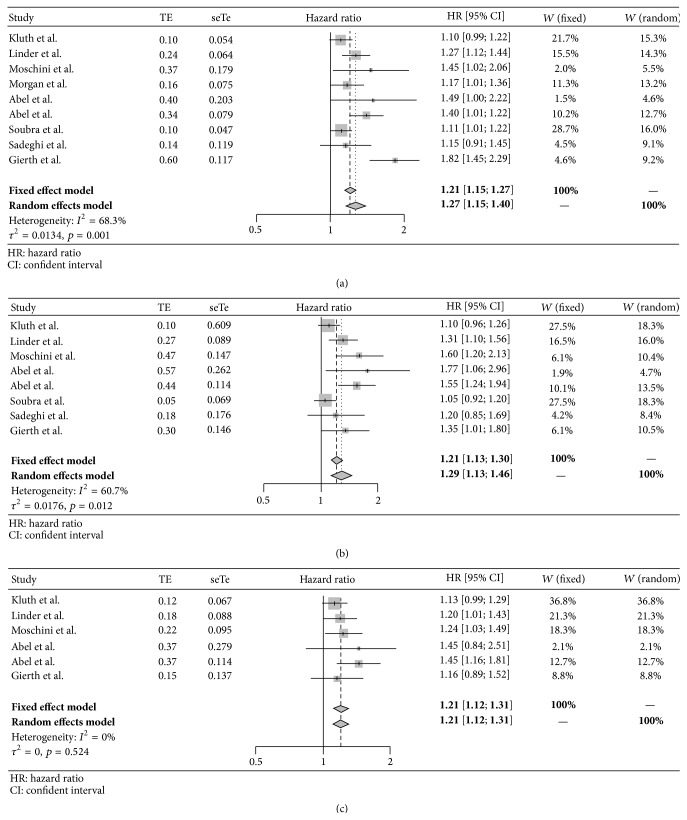
(a) Forrest plot for overall survival. (b) Forrest plot for cancer-specific survival. (c) Forrest plot for recurrence-free survival. HR: hazard ratio. CI: confidence interval.

Eight studies including a total of 15,655 patients reported overall mortality as an outcome (Table 1(a)). Of those patients, 38% (*n* = 5,940) received allogeneic BT during and/or after surgery. A negative impact of blood transfusions was found in 6 studies. The 2 studies that did not identify BT as an independent risk factor of OS did observe an important trend to worse OS [9, 10]. As shown in Figure 2, perioperative BTs were associated with a 27% (OR [95% CI]: 1.27 [1.15–1.40], *p* < 0.05) increased risk in mortality (Figure 2(a)). The *I* ^2^ test demonstrated moderate to substantial heterogeneity (68.3%, *p* = 0.0014) across the studies.

### 3.3. Cancer-Specific Survival

**Figure 3:**
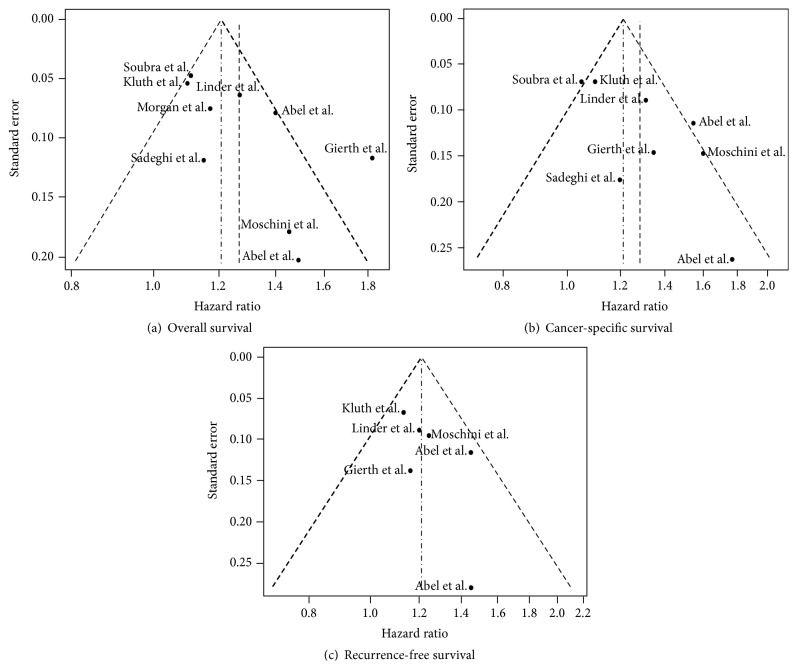
Funnel plots for outcomes.

Seven studies including a total of 14,878 patients estimated cancer-specific survival in the statistical analysis (Table 1(b)). The rate of transfusion in this pool of patients was 38% (*n* = 5,618). Five of the 7 studies (*n* = 6,521) demonstrated a negative impact of BT. As shown in Figure 3, the risk of dying from cancer after perioperative BT was 29% (OR [95% CI]: 1.29 [1.13–1.46], *p* < 0.05) (Figure 2(b)). The *I* ^2^ test demonstrated moderate heterogeneity (60%, *p* = 0.012) across the studies.

### 3.4. Recurrence-Free Survival

Five studies including a total of 8,778 patients estimated recurrence-free survival (Table 1(c)). Forty-eight percent (*n* = 4,270) of the patients received BTs. Three of the 5 studies (*n* = 4,910) showed a significant association between perioperative blood transfusions and poor survival [10, 17, 20]. In Abel's study patients the association was present for the Mayo Clinic's population of patients but not for University of Wisconsin's patients. As shown in Figure 2, perioperative BTs were associated with a significant increased risk in reduced RFS (OR [95% CI]: 1.12 [1.12–1.31], *p* < 0.05) (Figure 2(c)). The *I* ^2^ test demonstrated low heterogeneity (0%, *p* = 0.549) across the studies.

### 3.5. Sensitivity Analysis

The sensitivity analysis demonstrated that none of the studies included in the meta-meta-analysis was very influential, as the HR ranged from 1.20 to 1.30, 1.24 to 1.34, and 1.18 to 1.26 for OS, CSS, and RFS, respectively, for the pooled meta-analysis and all omitted meta-analyses (Table 2).

## 4. Discussion

Both the true nature of an association between BT and cancer recurrence and the biologic mechanism to explain this association are still very much unanswered research questions. The most commonly cited and investigated mechanism is the one that involves immune suppression or TRIM [11]. However, it has been speculated that the infusion of growth factors (vascular endothelial growth factor and transforming growth factor-b) and an enhanced inflammatory response as a result of the exposure of the recipient immune system to donor microparticles could also stimulate spread and proliferation of cancer cells [24, 25]. The present meta-analysis was not designed to investigate these possibilities; however, our results support the hypothesis that the perioperative administration of pRBCs is an independent risk factor for reduced RFS, CSS, and OS after RC for bladder cancer similar to what has been reported for other cancers such as colon, lung, and esophagus [12, 26–28].

Although a recent meta-analysis conducted by Wang and colleagues showed similar results to ours, we consider the findings of the present study clinically relevant because first we included data from two recently published cohort studies with relatively large sample size. Therefore, a larger number of transfused and not transfused patients were part of the pooled analysis of the present meta-analysis. And second we conducted a different analysis (random effects and fixed effect models) in comparison to that published by Wang and colleagues who used a fixed model paradigm [15]. We believe that a random effects model strengthens the analysis and adds significant information to the current evidence because this model assumes that the pooled studies are not functionally equivalent as they were conducted by researchers operating independently. Sources of variation among the studies used in the meta-analysis are, for instance, time of transfusion (intra- versus post- versus intra- and postoperative) and trigger of transfusions. Therefore, our analysis can be generalized to different clinical scenarios of bladder cancer surgery [29, 30]. It is worth mentioning that two studies did try to evaluate the impact of time of transfusion on outcomes and found that intraoperative BTs are an independent risk factor for poor survival while postoperative BTs do not show an association with worse outcomes [10, 17].

Our meta-analysis shows significant heterogeneity or high degree of dissimilarity among studies for CSS and OS but not for RFS. Although the high level of heterogeneity between studies for CSS and OS tempers the strength of any conclusions that can be made about the effect of BT on these two survival outcomes, the low heterogeneity and identical estimated HRs for RFS using both random effects and fixed effect models suggest a strong association between perioperative BT and BC recurrence after RC. In this meta-analysis, patients who received a perioperative BT had a 21% higher risk of BC recurrence than patients who did not receive BT.

The present study has the limitations inherent to any study level meta-analysis of cohort studies. Although we used the Ottawa-Newcastle score to grade study quality, all of the included studies were retrospective; the possibility exists that confounding variables (i.e., staging and tumor volume) may have influenced the individual study results and by extension the findings of this meta-analysis. Furthermore, the results of the present study cannot be extrapolated to the use of* autologous* blood transfusion since it is assumed that all studies included in the meta-analysis reported outcomes in patients transfused with mainly allogeneic blood.

In conclusion, perioperative BT may be associated with reduced RFS, CSS, and OS in patients undergoing RC for BC. A well-designed prospective RCT is needed in this population to provide the high level evidence necessary for answering this question.

## Figures and Tables

**(a):** 

Author	Year	Ottawa-Newcastle score	*N* = total	*N* = transfused patients	*N* = nontransfused patients	Hazard ratio	95% CI for HR
Kluth et al. [9]	2014	8.00	2895	1128	1767	1.1	0.99–1.22
Linder et al. [20]	2013	9.00	2060	1279	781	1.27	1.12–1.45
Moschini et al. [17]	2015	7.00	1373	463	910	1.45	1.02–2.08
Morgan et al. [21]	2013	7.00	777	323	454	1.17	1.01–1.36
Abel et al. [10]	2014	7.00	281	162	119	1.49	1.00–2.25
Abel et al. [10]	2014	7.00	1485	815	670	1.4	1.2–1.62
Soubra et al. [18]	2015	8.00	5462	1139	4323	1.109	1.011–1.21
Sadeghi et al. [22]	2012	7.00	638	209	429	1.15	0.91–1.45
Gierth et al. [23]	2015	7.00	684	423	261	1.822	1.45–2.29

**(b):** 

Author	Year	Ottawa-Newcastle score	*N* = total	*N* = transfused patients	*N* = nontransfused patients	Hazard ratio	95% CI for HR
Kluth et al. [9]	2014	8.00	2895	1128	1767	1.1	0.96–1.27
Linder et al. [20]	2013	9.00	2060	1279	781	1.31	1.1–1.57
Moschini et al. [17]	2015	7.00	1373	463	910	1.6	1.2–2.26
Abel et al. [10]	2014	7.00	281	162	119	1.77	1.06–2.94
Abel et al. [10]	2014	7.00	1485	815	670	1.55	1.24–1.94
Soubra et al. [18]	2015	8.00	5462	1139	4323	1.052	0.919–1.204
Sadeghi et al. [22]	2012	7.00	638	209	429	1.2	0.85–1.69
Gierth et al. [23]	2015	7.00	684	423	261	1.35	1.015–1.795

**(c):** 

Author	Year	Ottawa-Newcastle score	*N* = total	*N* = transfused patients	*N* = nontransfused patients	Hazard ratio	95% CI for HR
Kluth et al. [9]	2014	8.00	2895	1128	1767	1.13	0.99–1.28
Linder et al. [20]	2013	9.00	2060	1279	781	1.20	1.01–1.42
Moschini et al. [17]	2015	7.00	1373	463	910	1.24	1.03–1.65
Abel et al. [10]	2014	7.00	281	162	119	1.45	0.84–2.51
Abel et al. [10]	2014	7.00	1485	815	670	1.45	1.16–1.81
Gierth et al. [23]	2015	7.00	684	423	261	1.16	0.886–1.519

CI: confidence interval. HR: hazard ratio.

**Table 2:** Sensitivity analysis.

Study	Study influence analysis OS^*∗*^				Study influence analysis CSS^*∗∗*^				Study influence analysis RFS^*∗∗∗*^			
	HR	95% CI for HR	*τ* ^2^	*I* ^2^, %	HR	95% CI for HR	*τ* ^2^	*I* ^2^, %	HR	95% CI for HR	*τ* ^2^	*I* ^2^, %
Omitting Kluth et al. [9]	1.30	1.16–1.45	0.0151	67.5	1.34	1.15–1.55	0.021	59.8	1.26	1.14–1.39	0	0
Omitting Linder et al. [20]	1.27	1.13–1.42	0.0168	71.4	1.29	1.11–1.49	0.0227	64.6	1.22	1.11–.133	0.0005	3.9
Omitting Moschini et al. [17]	1.26	1.13–1.39	0.0137	71.0	1.25	1.10–1.41	0.0144	57.3	1.21	1.10–1.32	0.0003	2.4
Omitting Morgan et al. [21]	1.29	1.15–1.44	0.0164	72.1	NA	NA	NA	NA	NA	NA	NA	NA
Omitting Abel et al. [10]	1.26	1.14–1.39	0.0136	71.0	1.26	1.11–1.43	0.0163	61.7	1.26	1.14–1.39	0	0
Omitting Abel et al. [10]	1.25	1.12–1.38	0.0128	67.1	1.24	1.10–1.40	0.0128	52.8	1.22	1.11–.133	0.0005	3.9
Omitting Soubra et al. [18]	1.30	1.16–1.45	0.0153	66.4	1.34	1.18–1.52	0.0137	49.3	NA	NA	NA	NA
Omitting Sadeghi et al. [22]	1.28	1.15–1.43	0.0149	72.1	1.30	1.13–1.49	0.0205	66.3	NA	NA	NA	NA
Omitting Gierth et al. [23]	1.20	1.12–1.30	0.0043	42.4	1.28	1.12–1.47	0.0201	65.2	1.21	1.10–1.32	0.0003	2.4

^*∗*^The summary HR when all studies are included was 1.27 (95% CI: 1.15, 1.40) with *τ* ^2^ = 0.0134, *I* ^2^ = 68.3%. The HR of transfusion ranged from 1.20 to 1.30 for the pooled meta-analysis and all omitted meta-analyses.

^*∗∗*^The summary HR when all studies are included was 1.29 (95% CI: 1.13, 1.46) with *τ* ^2^ = 0.0176, *I* ^2^ = 60.7%. The HR of transfusion ranged from 1.24 to 1.34 for the pooled meta-analysis and all omitted meta-analyses.

^*∗∗∗*^The summary HR when all studies are included was 1.21 (95% CI: 1.12, 1.31) with *τ* ^2^ = 0, *I* ^2^ = 0%. The HR of transfusion ranged from 1.18 to 1.26 for the pooled meta-analysis and all omitted meta-analyses.

## Patient Summary

In this report we looked at the survival outcomes of patients with bladder cancer who received blood transfusion during and/or after surgery for their malignant disease. This study pooled and aggregated data from other published studies. We found that blood transfusions are associated with worse survival.
